# IGF1R activation and the in vitro antiproliferative efficacy of IGF1R inhibitor are inversely correlated with *IGFBP5* expression in bladder cancer

**DOI:** 10.1186/s12885-017-3618-5

**Published:** 2017-09-07

**Authors:** Yann Neuzillet, Elodie Chapeaublanc, Clémentine Krucker, Leanne De Koning, Thierry Lebret, François Radvanyi, Isabelle Bernard-Pierrot

**Affiliations:** 1 Hôpital Foch, Département d’Urologie, 40 Rue Worth, 92151 Suresnes, France; 2 Université de Versailles – Saint-Quentin-en-Yvelines, 78000 Versailles, France; 3 Institut Curie, PSL Research University, CNRS, UMR144, Equipe Labellisée Ligue contre le Cancer, 75005 Paris, France; 4 Sorbonne Universités, UPMC Université Paris 06, CNRS, UMR144, 75005 Paris, France; 5 Département de Recherche Translationnelle, Cedex 05, 75248 Paris, France; 6 UMR 144 CNRS/IC, Institut Curie, 26 rue d’Ulm, CEDEX 05, 75248 Paris, France

**Keywords:** Bladder cancer, Oncogenesis, IGF, IGFR, IGFBP, IGF1R inhibitor

## Abstract

**Background:**

The insulin growth factor (IGF) pathway has been proposed as a potential therapeutic target in bladder cancer. We characterized the expression of components of the IGF pathway — insulin growth factor receptors (INSR, IGF1R, IGF2R), ligands (INS, IGF1, IGF2), and binding proteins (IGFBP1–7, IGF2BP1–3) — in bladder cancer and its correlation with IGF1R activation, and the anti-proliferative efficacy of an IGF1R kinase inhibitor in this setting.

**Methods:**

We analyzed transcriptomic data from two independent bladder cancer datasets, corresponding to 200 tumoral and five normal urothelium samples. We evaluated the activation status of the IGF pathway in bladder tumors, by assessing IGF1R phosphorylation and evaluating its correlation with mRNA levels for IGF pathway components. We finally evaluated the correlation between inhibition of proliferation by a selective inhibitor of the IGF1R kinase (AEW541), reported in 13 bladder cancer derived cell lines by the Cancer Cell Line Encyclopedia Consortium and mRNA levels for IGF pathway components.

**Results:**

IGF1R expression and activation were stronger in non-muscle-invasive than in muscle-invasive bladder tumors. There was a significant inverse correlation between IGF1R phosphorylation and *IGFBP5* expression in tumors. Consistent with this finding, the inhibition of bladder cell line viability by IGF1R inhibitor was also inversely correlated with *IGFBP5* expression.

**Conclusion:**

The IGF pathway is activated and therefore a potential therapeutic target for non muscle-invasive bladder tumors and IGFBP5 could be used as a surrogate marker for predicting tumor sensitivity to anti-IGF therapy.

**Electronic supplementary material:**

The online version of this article (10.1186/s12885-017-3618-5) contains supplementary material, which is available to authorized users.

## Background

Bladder cancer is the 9th most common cancer diagnosis worldwide [1]. At first diagnostic, 70% of cases do not infiltrate the bladder muscle (non-muscle-invasive bladder cancer, NMIBC). Cystoscopy and transurethral resection of the tumor are the mainstays for the diagnosis and initial treatment of NMIBC. Adjuvant intravesical instillation therapies may be recommended, depending on the clinical and pathological features of the tumor, to decrease the risks of cancer recurrence and, in certain circumstances, tumor progression. Nevertheless, the overall efficacy of such treatments is limited and these risks remain a matter of concern. The cancer eventually recurs in about 50% of NMIBC cases, and overall 10% progress to muscle-invasive disease with 50% of cases in high risk group [2]. Despite radical cystectomy as standard treatment for localized muscle-invasive bladder cancer (MIBC) [3], the survival at five years is only about 50% of patients [4] and the 5-year survival is less than 20% in case of distant metastases [5]. There is therefore a major need to identify more effective agents for treating bladder cancer, either alone or in combination with established drugs in particular for muscle-invasive and/or metastatic tumors.

Insulin and insulin-like growth factors (IGFs) are known to regulate energy metabolism and growth. There are now considerable evidences for an important role of these hormones and the signal transduction networks they regulate in oncogenesis [6]. The IGF family consists of three peptide ligands (INS, IGF1, and IGF2), three specific cell surface receptors (INSR, IGF1R, and IGF2R), and ten specific IGF-binding proteins (IGFBP1 to IGFBP7 and IGF2BP1 to IGF2BP3) [7]. The mitogenic effects of IGFs are mediated principally through interactions with IGF1R, the key node for IGF pathway signaling but also by the insulin receptor. One of the functions of IGFBPs is to compete with cell surface receptors for free IGF1 and IGF2, thereby controlling the bioavailability of IGFs in target cells. IGF1R was reported to be overexpressed in muscle-invasive bladder cancer and associated with outcome in these tumors [8, 9]. Furthermore, IGF1R was involved in bladder cancer cell motility and invasion in the presence of an exogenous ligand suggesting that the IGF1R might play a critical role in the establishment of the invasive phenotype in urothelial neoplasia [10]. Proline-rich tyrosine kinase 2 (PTK2B), which is strongly activated by IGF1, has been shown to be critical for IGF1R-dependent motility and invasion and for regulation of the IGF1-dependent activation of AKT and MAPK pathways [11]. Patients with bladder cancer also display a significant increase in urinary IGF2 concentration [12]. Thus, activation of the IGF1R/INSR pathway may involve mechanisms different from overexpression of the receptor, such as autocrine stimulation due to the overproduction of IGF2.

We investigated here IGF pathway activation in bladder cancer, to evaluate the potential utility of this pathway as a therapeutic target in this cancer. Clinical trials of anti-IGF1R treatment without selection of patient have yielded disappointing results, highlighting the importance of surrogate markers for predicting IGF pathway activation and drug sensitivity [13]. We therefore first characterized the expression of genes encoding proteins involved in the IGF pathway in bladder tumor samples. We further studied the expression of IGF1R and its phosphorylated form in these samples, using reverse-phase protein arrays. We then investigated the possible correlation between mRNA levels for components of the IGF pathway and IGF1R phosphorylation levels. Finally, using publicly available data from the Cancer Cell Line Encyclopedia (http://www.broadinstitute.org/ccle), we assessed the correlation between mRNA levels for components of the IGF pathway and cell sensitivity (inhibition of cell viability) to a tyrosine kinase inhibitor directed against IGF1R in bladder cancer-derived cell lines.

## Methods

### Samples

We analyzed transcriptomic data (coding RNA) data from two independent bladder cancer datasets (FBLAD-U95 and FBLAD-Exon). The FBLAD-U95 set of 75 bladder tumors (24 Ta, 12 T1, and 39 *T* ≥ 2 tumors) was obtained from 75 patients included between 1988 and 2001 at the Department of Urology of Henri Mondor Hospital (Créteil, France). The FBLAD-Exon set of 125 bladder tumors (24 Ta, 32 T1, and 69 *T* ≥ 2 tumors) was obtained from patients included between 1993 and 2006 at the Department of Urology of Foch Hospital (*n* = 45) (Suresnes, France), Henri Mondor Hospital (*n* = 52) (Créteil, France), and Institut Gustave Roussy (*n* = 28) (Villejuif, France). The characteristics of the patients and the tumors in the two sets are summarized in Table 1. All patients provided written informed consent and the study was approved by the ethics committees of the different hospitals. Five normal urothelial samples were also used for transcriptomic analysis. They were obtained from fresh urothelial cells scraped from the normal bladder wall and dissected from the lamina propria during organ procurement from a cadaveric donor for transplantation.

**Table 1:** Patient and tumor characteristics

	FBLAD-U95 set	FBLAD-Exon set
Patients	*n* = 75	*n* = 125
Sex		
Male, *n* (%)	61 (81.3)	100 (80.0)
Female, *n* (%)	14 (18.7)	25 (20.0)
Mean age at surgery, years ± SD	62.8 ± 13.8	69.7 ± 15.2
Mean follow-up, months ± SD	40.1 ± 40.0	33.6 ± 29.7
Bladder tumors		
Clinical presentation		
Incident tumors, *n* (%)	58 (77.3)	107 (85.6)
Recurrent tumors, *n* (%)	17 (22.7)	18 (14.4)
TNM 2009 Stage		
Ta, *n* (%)	24 (32.0)	24 (19.2)
T1, *n* (%)	12 (16.0)	32 (25.6)
*T* ≥ 2, *n* (%)	39 (52.0)	69 (55.2)
WHO 1973 Grade		
G1, *n* (%)	11 (14.7)	5 (4.0)
G2, *n* (%)	23 (30.7)	26 (20.8)
G3, *n* (%)	41 (54.7)	94 (75.2)

### Extraction of RNA, DNA and protein from tissues

Immediately after surgery, the samples were frozen in liquid nitrogen and stored at −80 °C until nucleic acid and protein extraction. RNA, DNA, and proteins were extracted from the surgical samples by cesium chloride density centrifugation. Briefly, the frozen samples were homogenized in 4 M guanidium thiocyanate, with an Ultraturax T25 homogenizer (Janke & Kunkel, IKA-Labortechnik, Staufen, Germany). The homogenate was then centrifuged on a 5.7 M cesium chloride cushion. The RNA was found in the pellet, whereas the DNA was found on top of the cesium chloride cushion and proteins were found in the upper layer. The RNA and DNA were further purified by phenol–chloroform extraction and ethanol precipitation, and the proteins were dialyzed and lyophilized. The concentration, integrity and purity of each RNA sample were determined with the RNA 6000 LabChip Kit (Agilent Technologies, Massy, France) and an Agilent 2100 bioanalyzer. DNA purity was also assessed by determining the ratio of absorbances at 260 and 280 nm. DNA concentration was determined with a Hoechst dye-based fluorescence assay. Protein dialysis was performed at 4 °C for 24 h with a 2–4 kDa cutoff dialysis membrane and 0.1 M sodium bicarbonate buffer (pH 8.2). The dialyzed proteins were freeze-dried and then suspended in boiled Laemmli buffer without bromophenol blue (50 mM Tris pH =6.8, 2% SDS, 5% glycerol, 2 mM DTT, 2.5 mM EDTA, 2.5 mM EGTA, 1× HALT phosphatase inhibitor (Perbio), MINI EDTA-free Complete protease inhibitor cocktail (Roche, 1 tablet/10 mL), 2 mM Na_3_VO_4_ and 10 mM NaF) and boiled for 30 min. Protein concentration was evaluated in a reducing agent-compatible BCA test (Life technologies, Saint-Aubin, France).

### Affymetrix array data

For the FBLAD-U95 set, we used the Human Genome U95A and U95Av2 arrays (Affymetrix) containing almost 12,500 probe sets. Data were available from Stransky et al. [14] (E-TABM-147). For the FBLAD-Exon set, the Human Genome Exon 1.0ST arrays (Affymetrix) containing almost 289,961 probe sets were used. RNA amplification, cDNA probe labeling and hybridization were performed as described on the Affymetrix website. The Affymetrix DNA microarray results were normalized with RMA (robust multi-array averaging) algorithm [15]. The BrainArray annotation was used [16]. BrainArray annotation ENTREZG (version 12, available at http://brainarray.mbni.med.umich.edu/Brainarray/Database/CustomCDF/CDF_download.asp#v12) provided one remapped probeset per gene, according to National Center for Biotechnology Information (NCBI) *Homo sapiens* ENTREZGENE build 36.1.We focused on 16 genes encoding members of the IGF family receptors (*INSR*, *IGF1R*, and *IGF2R*), ligands (*INS*, *IGF1*, and *IGF2*), and binding proteins (*IGFBP1*, *IGFBP2*, *IGFBP3*, *IGFBP4*, *IGFBP5*, *IGFBP6*, *IGFBP7*, *IGF2BP1*, *IGF2BP2*, and *IGF2BP3*), referred here to collectively as components of the IGF pathway. Data relative to these genes are summarized in Additional file 1: Table S1.

### Reverse-phase protein array (RPPA)

RPPA was performed and analyzed as previously described [17]. Briefly, samples were deposited on nitrocellulose-covered slides (Schott Nexterion NC-C, Jena, Germany) with a dedicated arrayer (2470 Arrayer, Aushon Biosystems, Billericay, MA, USA). Four serial dilutions, at concentrations ranging from 1500 to 187 μg/mL, and four technical replicates per dilution were used for each sample. Arrays were incubated with specific anti-IGF1Rβ (not reactive with insulin receptor) (#3027) or anti-phosphorylated insulin receptor β (TYR1361)/ phospho-IGF1Rβ (#3023) (Cell Signaling Technology, Ozyme, France) antibodies or without primary antibody (negative control), in an Autostainer Plus (Dako, Trappes, France). The slides were first incubated with avidin, biotin and peroxidase blocking reagents (Dako, Trappes, France) before saturation with TBS containing 0.1% Tween-20 and 5% BSA (TBST-BSA). They were then probed by incubation overnight at 4 °C with primary antibodies diluted in TBST-BSA. They were washed in TBST and probed by incubation with horseradish peroxidase-coupled secondary antibodies (Jackson ImmunoResearch Laboratories, Newmarket, UK) diluted in TBST-BSA for 1 h at room temperature. The signal was amplified by incubating the slides with Bio-Rad Amplification Reagent for 15 min at room temperature. The arrays were washed with TBST, incubated with Alexa647-streptavidin (Molecular Probes) diluted in TBST-BSA for 1 h at room temperature and washed again in TBST. For total protein staining, arrays were incubated for 15 min in 7% acetic acid and 10% methanol, rinsed twice in water, incubated for 10 min in Sypro Ruby (Invitrogen, Cergy Pontoise, France) and rinsed again. The processed slides were dried by centrifugation and scanned with a GenePix 4000B microarray scanner (Molecular Devices, Sunnyvale, USA). Spot intensity was determined with MicroVigene software (VigeneTech Inc., Carlisle, USA), corrected for the background signal obtained in the absence of antibody and normalized against the Sypro Ruby signal. The antibodies used for RPPA were tested by western blotting before use, to assess their specificity for the protein of interest within 18 tumor lysates and presented Pearson correlation coefficient between RPPA and western blotting greater than 0.7 for both antibodies used (data not shown). RPPA signal obtained with 18 bladder tumor samples using anti-phospho-INSR/IGF1R (#3023) antibody was also correlated with western-blot signal of an anti-phospho-IGFR1 (TYR1316)(#6113) (not specific enough to be used for RPPA, cross reacts with other tyrosine kinase receptor but not with INSR) (Cell Signaling Technology, Ozyme, France) (Pearson *r* = 0.68) (data not shown) suggesting that RPPA phosphorylated-INSR/IGF1R signal is proportional to IGF1R phosphorylation in bladder tumors.

### Cell line

RT112 bladder cancer-derived cell line was obtained from DKFZ (Heidelberg, Germany) and cultured in Dulbecco’s modified Eagle’s medium F-12 (Invitrogen, Cergy Pontoise, France) supplemented with 10% fetal bovine serum. The identity of the cell line used was checked with comparative genomic hybridization arrays, assessed with BAC arrays, and *FGFR3* and *TP53* mutations which were investigated with the SNaPshot technique (for *FGFR3*) or classical sequencing (for *TP53*). FGFR3-TACC3 fusion was confirmed by PCR.

### Assessment of cell viability after IGF1R inhibition or depletion

Transient transfections were performed in 24-well plates in the presence of Lipofectamine RNAimax, used according to the manufacturer’s instructions (Invitrogen, Cergy Pontoise, France), with 20 nmol/L siRNA. A negative control siRNA and a pre-validated siRNA specific for IGF1R were purchased from Ambion. Neutralizing antibody experiments were performed with a mouse monoclonal antibody directed against human IGF1R (R&D Systems). We dispensed 5000 cells into each of the wells of a 96-well plate and incubated the plates for 24 h. The cells were then incubated for 72 h with DMEM (containing 1% serum) containing 10 μg/ml anti-IGF1R, or mouse IgG (10 μg/ml; R&D Systems) as a negative control. We determined cell viability in a colorimetric MTT assay performed 72 h after transfection or after the addition of anti-IGF1R antibody. All the experiments were performed in triplicate and were carried out at least three times.

### Publicly available data for the sensitivity of bladder cell lines to IGF1R inhibitor

We evaluated the effect on cell viability of an inhibitor of IGF1R kinase AEW541 [18], on 13 bladder cancer-derived cell lines (5637, HT1197, HT1376, J82, JMSU1, KMBC2, RT4, RT112, SCaBER, SW780, T24, TCCSUP and UMUC3), using Broad-Novartis Cancer Cell Line Encyclopedia (CCLE) collaborative project data [19] (http://www.broadinstitute.org/ccle). For simultaneous assessments of the efficacy and potency of a drug, they designated an ‘activity area’, defined as the area above the curve of relative cell viability inhibition against drug concentration. The higher the activity area is, the higher the cell sensitivity to the inhibitor is. All cell lines were characterized with several genomic technology platforms, including Affymetrix U133 plus 2.0 arrays for the assessment of mRNA levels.

### Statistical analyses

Linear Models for Microarray Data (LIMMA) was used to analyze complex experiments involving simultaneous comparisons between large numbers of RNA targets [20]. Non-parametric Spearman’s rank correlation tests were carried out to evaluate the correlation between the phosphorylated IGF1R signal or anti-IGF1R sensitivity and levels of mRNA for the components of the IGF pathway. All functional experiments were carried out twice or three times, in triplicate. Data are expressed as means ± SD. Student’s *t-*tests were used for the statistical analysis. The control siRNA group or the IgG group was used as the reference group.

## Results

### Changes in the expression of genes encoding components of the IGF/IGFR system in bladder cancer

**Fig. 1:**
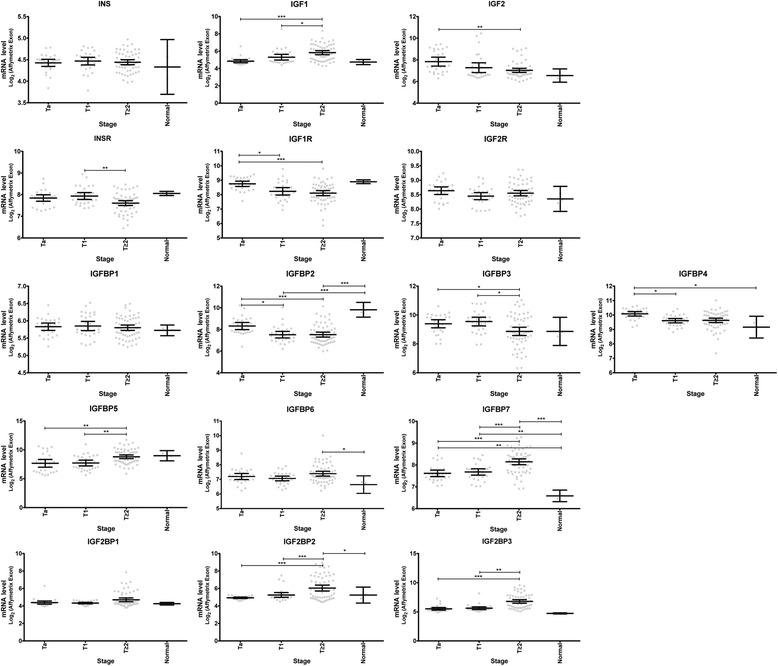
Characterization of the expression of genes encoding proteins involved in the IGF pathway points out dysregulation of IGF pathway in bladder tumors. Levels of mRNA for IGF receptors, ligands, and binding proteins (Log_2_ scale), according to tumor stage, for the FBLAD-Exon dataset. *: *p* value = 0.01 to 0.05 **: *p* value = 0.001 to 0.01 ***: *p* value ≤0.001

We studied changes in the level of expression of 16 genes of the IGF pathway (IGF receptors, ligands, and binding proteins) during bladder tumor progression. We carried out LIMMA tests, to compare mRNA levels between tumors of different stages, from two independent datasets (FBLAD-Exon and FBLAD-U95 datasets corresponding to 125 and 75 tumors, respectively, see Table 1). We report the *p*-values obtained in Table 2. Transcriptomic data for IGF pathway genes during tumor progression are shown in Fig. 1 for the FBLAD-Exon dataset, and in Additional file 2: Figure S1 for FBLAD-U95 dataset. We searched for genes displaying the same pattern of significant change in expression in both series (Table 2). The levels of expression of IGF receptors and ligands genes did not differ significantly between cancerous and normal urothelium. However, the levels of expression of *IGF1R* and *IGF2* were lower in more invasive tumors (T1 and *T* ≥ 2). *IGFBP2*, *IGFBP3*, and *IGFBP4* were also significantly less strongly expressed in more invasive tumors. By contrast, *IGFBP7* was more strongly expressed in the tumors than in the normal samples, and in more invasive than less invasive tumors. Thus, neither IGF receptors nor ligands were expressed more strongly in more invasive tumors. However, some changes were observed in the levels of expression of binding protein genes, with a decrease in *IGFBP2*, *IGFBP3*, and *IGFBP4* expression and an increase in *IGFBP7* expression with tumor progression.

**Table 2:**
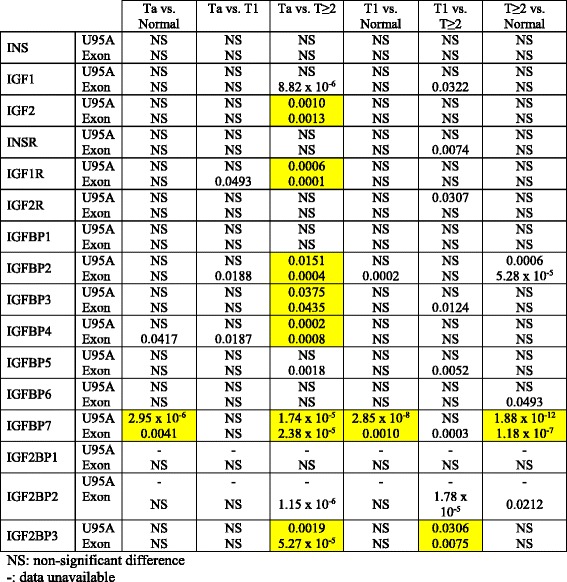
Affymetrix RMA signal comparisons in LIMMA tests

The boxes where the result is significant in both studies are highlighted

**Fig. 2:**
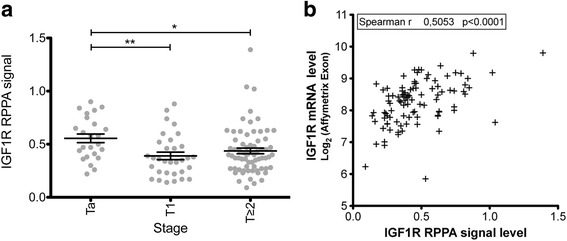
Levels of IGF1R mRNA and protein are stronger in less aggressive tumors. **a** RPPA signal for IGF1R by tumor stage; **b** correlation between IGF1R mRNA levels and IGF1R RPPA signal values in 97 tumors from the FBLAD-Exon dataset. *: *p* value = 0.01 to 0.05 **: *p* value = 0.001 to 0.01 ***: *p* value ≤0.001

Concerning IGF1R expression, our results being contradictory with previously published ones [9], we first studied levels of expression of IGF1R mRNA in others publicly available data for bladder tumors and we then sought to validate them at the protein level. Data for 19 normal and 211 muscle invasive bladder tumor samples from TCGA [21] (https://tcga-data.nci.nih.gov/docs/publications/tcga) and for 12 normal samples, 97 non muscle-invasive (NMIBC) and 45 muscle invasive bladder tumors (MIBC) from Lindgren et al., [22] also showed a significant down-regulation of the level of expression of IGF1R mRNA in MIBC as compared to normal samples and in MIBC as compared to NMIBC (Additional file 3: Figure S2). IGF1R protein levels were assessed by reverse-phase protein array (RPPA) analysis on a subset of 97 tumors of the FBLAD-Exon set. The IGF1R protein, like its mRNA, was present in significantly larger amounts in superficial tumors (Ta) than in more invasive tumors (T1 and *T* ≥ 2) (Fig. 2a). IGF1R protein levels on RPPA were also significantly correlated with the *IGF1R* mRNA levels obtained with the Affymetrix exon array for this subset of 97 samples from the FBLAD-Exon set (Fig. 2b). RPPA does not distinguish between protein expression in the tumor cells or in the stroma. To determine the type of cells expressing IGF1R in bladder tumors and to study IGF1R expression in normal urothelium, we took advantage of anti-IGF1R immunohistochemistry publically available thanks to Human Protein Atlas portal (http://www.proteinatlas.org/) [23]. In good agreement with two previously published studies using other anti-IGF1R antibodies [8, 9] (giving confidence on the selectivity of the antibodies used), results from Human Protein Atlas showed a membranous and cytoplasmic expression of IGF1R by epithelial tumor cells together with an absence of expression by stromal cells (Additional file 4: Figure S3a, right panel). Furthermore, in good agreement with our transcriptomic data, immunohistochemistry also revealed a strong expression of IGF1R by normal urothelial cells (Additional file 4: Figure S3a, left panel). Since IGF1R is not expressed by stromal cells but only by epithelial tumor cells, we compared the stromal infiltration of tumors with the highest and lowest expression of IGF1R, as assessed by RPPA, in our CIT-series for which HE staining are available [24]. We identified tumors that presented similar pattern of stromal infiltration but very different levels of IGF1R expression, some examples are shown in Additional file 4: Figure S3b. These results highly suggest that in our CIT-series of tumors, the difference of expression observed by RPPA is due to a different expression of IGF1R by tumor cells.

### Study of IGF1R activation, and its correlation with mRNA levels for IGFR pathway genes in bladder tumors

**Fig. 3:**
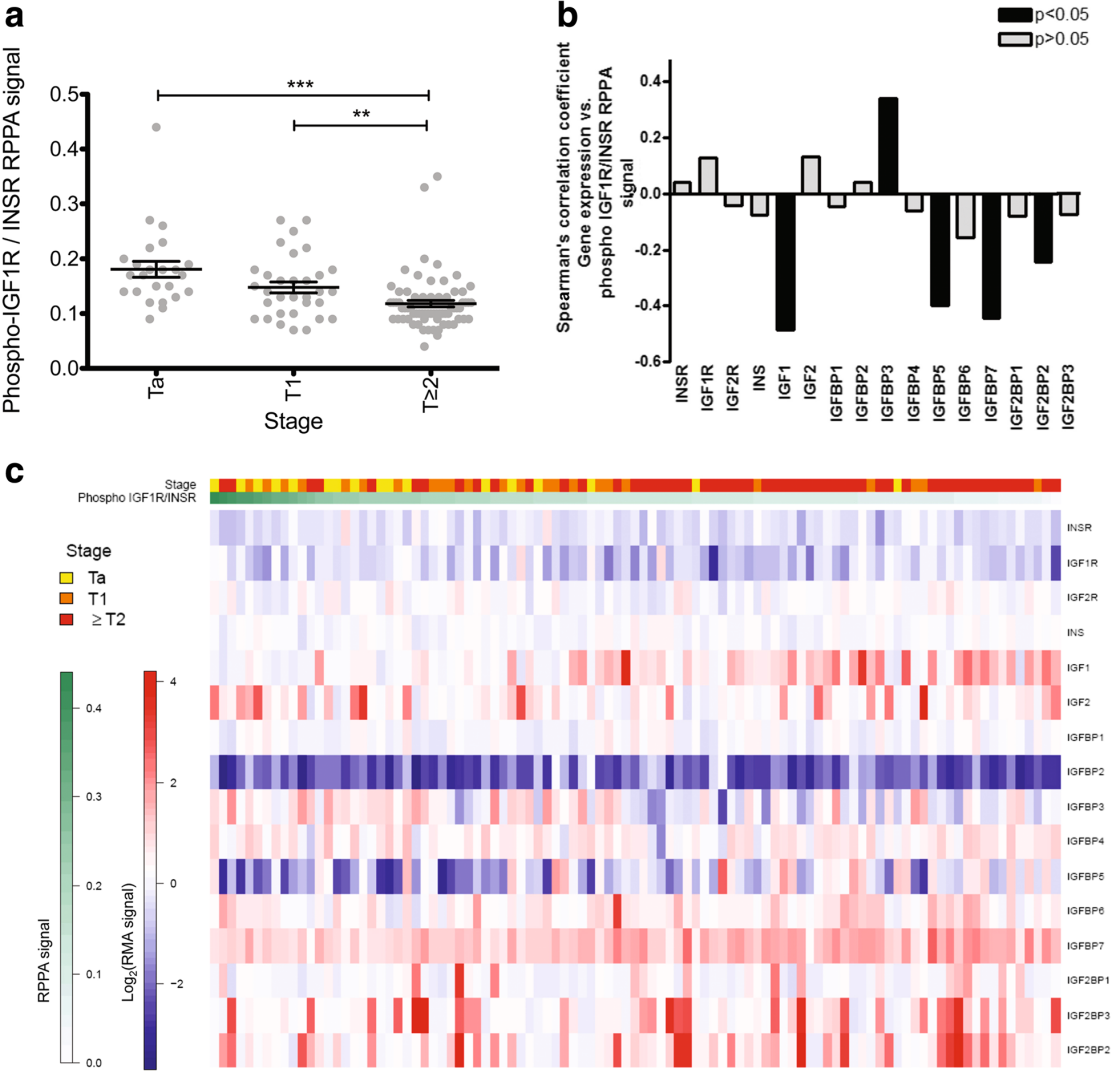
Activation of IGF pathway in bladder tumor is correlated with expression of some components of this pathway. **a** RPPA signal for phosphorylated IGF1R/INSR by tumor stages; **b** Spearman’s coefficient for the correlations between mRNA levels for IGF receptors, ligands, and binding proteins and the phosphorylated IGF1R/INSR RPPA signal; **c** heat map for the expression of IGF receptor, ligand, and binding protein genes in tumors, in decreasing order of phosphorylated IGF1R/INSR RPPA signal in the FBLAD-Exon dataset. Color intensity (blue-red scale) is centered on the mean value for expression in normal bladder samples

As IGF1R is the central node in IGF pathway signaling, we investigated IGF1R activation, by assessing the phosphorylation of this receptor. Only one antibody against phosphorylated-INSR/phosphorylated-IGF1R was suitable for RPPA. However RPPA signals obtained with this anti-phosphorylated INSR/IGF1R antibody for 18 bladder tumor samples were correlated with the western-blot signals obtained with an anti-phospho-IGFR1 antibody (Pearson *R* = 0.68) (see methods) strongly suggesting that anti- phospho INSR/IGF1R RPPA signal did reflect IGF1R phosphorylation in bladder tumor samples. We so further quantified INSR/IGF1R phosphorylation, by RPPA on 97 tumors from the FBLAD-Exon tumor set (Fig. 3a). The phosphorylated INSR/IGF1R RPPA signal was weaker for *T* ≥ 2 tumors than for Ta (0.11 ± 0.04 vs. 0.18 ± 0.08, *p* < 0.0001), and T1 tumors (0.11 ± 0.04 vs. 0.15 ± 0.05, *p* = 0.0033).

To identify predictor of IGF pathway activation within IGF member genes, we then evaluated the Spearman correlation between the phosphorylated-INSR/IGFR RPPA signal and the mRNA levels for IGF pathway genes (the Affymetrix Human Exon1.0 ST array signal) for these 97 bladder tumors from the FBLAD-Exon tumor set (Fig. 3b).

The phosphorylated INSR/IGF1R RPPA signal was significantly negatively correlated with *IGF1*, *IGFBP5*, *IGFBP7* and *IGF2BP2* mRNA levels (Fig. 3b and c), which were higher in more invasive tumors (Fig. 1, Table 2 and Additional file 2: Figure S1). Conversely, *IGFBP3* mRNA level was positively correlated with activated INSR/IGF1R levels, which were lower in more invasive tumors. Overall, activation of the INSR/IGF1R pathway was not correlated with mRNA levels for IGF receptors or IGF2. A heat map of gene expression levels in tumors, ordered in descending order, on the basis of the phosphorylated IGF1R signal, provided a visualization of the expression profile of IGF pathway genes in each tumor, and highlighted the mutually exclusive nature of IGF1 and IGF2 expression (Fig. 3c).

Thus, high levels of IGF1R and phosphorylated INSR/IGF1R expression were significantly associated with superficial tumors. In addition, the expression of several components of the IGF pathway was significantly positively correlated (*IGFBP3*) or inversely correlated (*IGF1*, *IGFBP5*, *IGFBP7*, *IGF2BP3*) with the activation of this pathway. Previous studies focused on the role of IGF1R in bladder cancer invasiveness. Our results indicated that this receptor was more likely to be activated in superficial tumors. We therefore evaluated its role in bladder cancer proliferation.

### Correlation of the inhibition of proliferation by a selective inhibitor of the IGF1R kinase (AEW541) and the expression of IGF receptor, ligand, and binding protein genes in the CCLE dataset

**Fig. 4:**
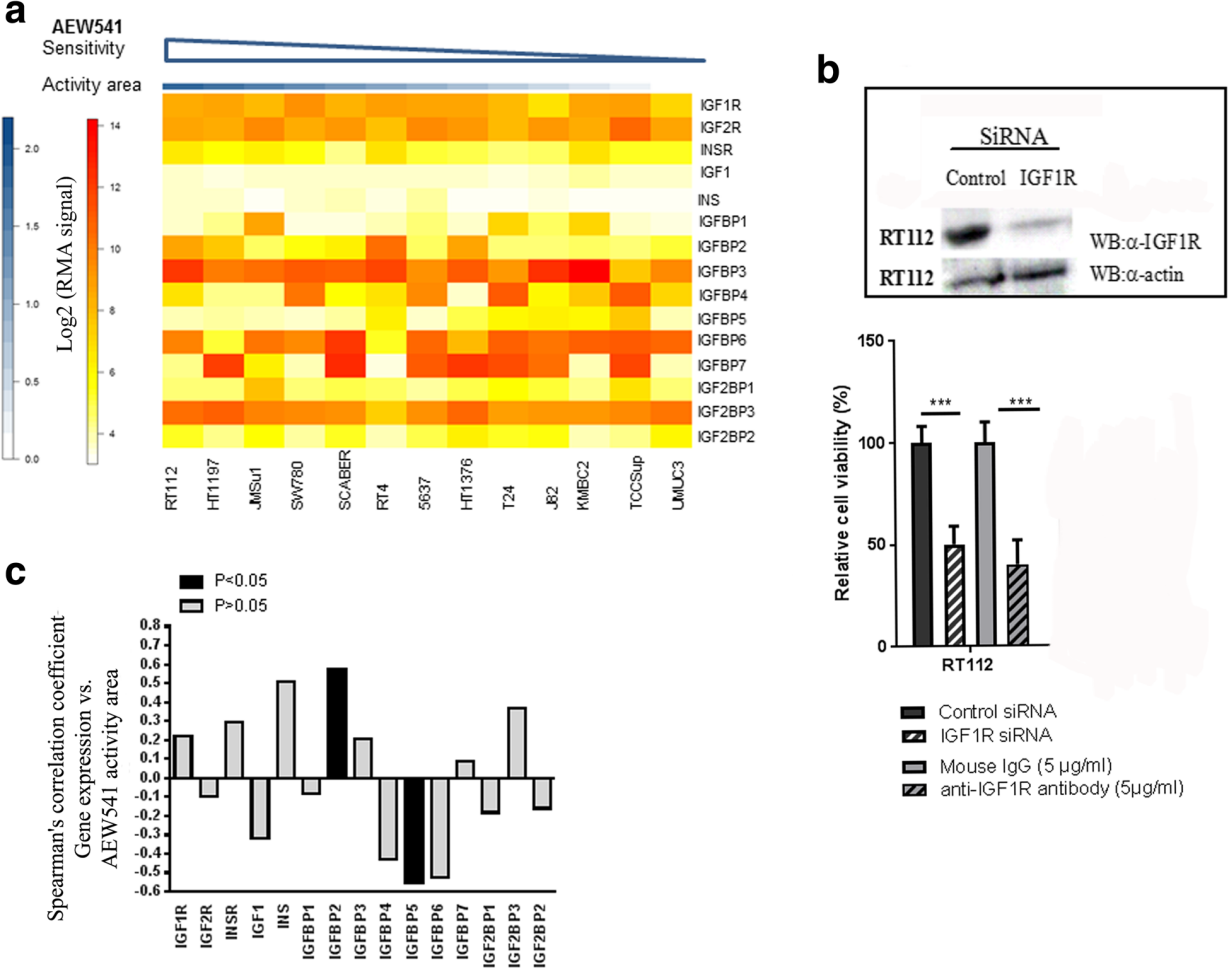
mRNA levels for IGFBP5 and IGFBP2 are biomarkers for bladder tumor cells sensitivity to IGF1R kinase inhibitor, AEW541. **a** Heat map of the IGF receptors, ligands, and binding proteins mRNA levels in regards to sensitivity to AEW41 in 13 bladder tumor derived cell lines. Data were extracted from CCLE database; **b** Effect of a blocking antibody against IGF1R and of IGF1R siRNA on the viability of RT112 cells. IGF1R knockdown 72 h after transfection with a control or anti-IGF1R siRNA was assessed by western blotting (inset). The effect of the siRNA on cell viability was assessed in MTT assays. The effect of the anti-IGF1R blocking antibody was assessed after 72 h, in MTT assays; **c** Spearman’s coefficients for the correlations between the sensitivity to AEW541 and mRNA levels for IGF receptors, ligands, and binding proteins, in 13 cell bladder tumor derived cell lines. Data were extracted from CCLE database

We took advantages of publicly available data from the Cancer Cell Line Encyclopedia (CCLE) project (http://www.broadinstitute.org/ccle) reporting sensitivity (assessed by measuring cell viability) to IGF1R kinase inhibitor (AEW541) in 13 bladder cancer-derived cell lines for which mRNA levels were also obtained with Affymetrix U133plus2.0 arrays DNA array. Cell lines were ordered in descending order of sensitivity to AEW541, as determined on the basis of activity area (see the methods section). RT112 cells were the most sensitive, and UMUC3 cells were the most resistant (Fig. 4a). The sensitivity of RT112 cells to IGF1R inhibition observed using AEW541 was confirmed with an anti-IGF1R blocking antibody or an anti-IGF1R siRNA decreasing IGF1R levels by 90% (Inset, Fig. 4b). The inhibition or loss of expression of IGF1R significantly decreased the viability of RT112 bladder cancer cells (60%) (Fig. 4b). Those results brought some confidence in AEW541 specificity towards IGF1R.

We then assessed the correlation between AEW541 sensitivity in the 13 bladder cancer cell lines and mRNA levels for the genes of the IGFR pathway (Fig. 4c). The mRNA levels for the genes considered are shown as a heat map (Fig. 4a). *IGFBP2* expression was significantly positively correlated with AEW541 sensitivity (Fig. 4c). Conversely, *IGFBP5* expression was significantly inversely correlated with this sensitivity (Fig. 4c). No other significant correlation was observed in this dataset, for the expression of any IGF receptor or ligand gene.

## Discussion

In this study, we characterized the levels of mRNAs encoding proteins involved in the IGF pathway in bladder cancers. We then assessed the correlation of these mRNA levels with tumor stage, activation of the INSR/IGF1R receptors (indicating activation of the IGF pathway) and the antiproliferative efficacy of an inhibitor of IGF1R tyrosine kinase.

We found that among the IGFBPs studied (IGFBP1–7), IGFBP7 is the only IGFBP down-regulated during tumor progression. This specificity is maybe linked to the fact that among these IGFBPs, IGFBP-7 is the only low affinity IGFs binding protein and is therefore more related to IGFBP8–10 that are part of the CCN family.

We found that IGF1R expression (mRNA and protein levels were) was higher in Ta tumors than in more invasive tumors and that *IGF1R* mRNA expression in normal urothelium did not differ significantly from that in tumor samples. These findings, confirmed at the mRNA level in two other publicly available data sets, go against the conclusions of the study by Rochester et al., the only other study to date to have considered IGF1R expression as a function of bladder cancer stage [9]. They found *IGF1R* mRNA and IGF1R levels to be significantly higher in MIBC tumors than in normal bladder tissues, consistent with an upregulation of transcription. The discrepancies observed may result from the number of samples studied. For the transcriptomic analysis, Rochester et al. have studied 15 normal urothelium samples and 17 MIBC samples, while we have studied a total of 11 normal samples and 368 MIBC samples in our study. For the protein analysis, discrepancy could also be due to very different techniques: heterogeneity within the tumor is difficult to assess by immunohistochemistry. On the other hand, RPPA does not distinguish between protein expression in the tumor cells or in the stroma.

At the protein level our results are more in agreement with Gonzalez-Roibon et al. study that reported an expression of IGF1R in 67% of MIBC as assessed by immuno-histochemical scoring of 100 MIBC using tissue micro-array (TMA) but no significant overexpression in tumors as compare to normal samples for 65 cases for which normal urothelium was also available [8].

This study is the first to show that the activation of the IGF pathway is stronger in Ta tumors than in more invasive tumors (T1 and *T* ≥ 2). The phosphorylation of INSR/IGF1R proteins, leads to the activation of two main signaling pathways: the PI3K-AKT/PKB pathway and the RAS-MAPK pathway. Interestingly, these two pathways are also activated by Fibroblast Growth Factor Receptor 3 (FGFR3) that is commonly mutated and constitutively activated in Ta tumors. The activation of these two receptors thus leads to the same proliferation signal, which is crucial in the oncogenesis of Ta G1/low-grade bladder cancers [25]. Consistent with this hypothesis, CCLE cell viability assays with the IGF1R kinase inhibitor (AEW541) on 13 bladder cancer cell lines showed that cell lines derived from low-grade/stage tumors and also presenting a translocated form of FGFR3 (RT112, SW780, RT4) were among the most sensitive to the anti-proliferative effects of AEW541 (Fig. 4a).

Finally, we used a supervised approached towards known IGF pathway’s gene to identify molecular predictors of IGFR pathway activation (measured as IGF1R phosphorylation) in tumors and sensitivity to an inhibitor of IGF1R tyrosine kinase (AEW541) in bladder derived cell lines. Surprisingly, our results demonstrate that the expression levels of genes encoding IGF receptors and ligands were not correlated with the activation of INSR/IGF1R receptors or with the anti-proliferative efficacy of AEW541. However, *IGFBP5* mRNA levels in bladder cancer samples and cell lines were negatively correlated with the activation of INSR/IGF1R, and in cell lines with the anti-proliferative efficacy of an IGF1R tyrosine kinase inhibitor, suggesting that IGFBP5 over-expression in MIBC might be a useful marker of non-sensitivity to IGF1R inhibitor. Using the same hypothesis-driven supervised approach focused on IGF pathway member expression, Pavlicek and al., also identified IGFBP5 as a biomarker of colon cancer cells sensitivity to another IGF1R tyrosine kinase inhibitor, Figitumumab [26]. Using an unsupervised approach that used the whole genome data, given the different kinds of cancers studied within the CCLE project, the sensitivity of cells lines to the IGFR kinase inhibitor AEW541 was also predicted by *IGFBP5* expression levels [19]. Thus, it may be possible to extrapolate the results reported here for bladder cancers to other cancer types [19]. At the opposite, others markers of IGFR inhibitor sensitivity identified previously [19, 26] such as MYB did not predict sensitivity to AEW541 in bladder tumor derived cell lines and seem so to be cancer specific (data not shown). IGFBP5 is a carrier protein that may increase the half-life and the turnover in the bloodstream of IGFs [27]. IGFBP5 can play various roles during tumor progression [28], sometimes in absence of IGFs, supporting the existence of some IGF-independent activities [29]. I*GFBP5* expression is altered in various cancers, including breast cancer, neuroblastoma, osteosarcoma, lung cancer, colon cancer, and its impact on prognosis has been shown to be cell type-dependent and tissue type-dependent [27]. Liang et al. recently showed that *IGFBP5* overexpression was associated with a poor prognosis in patients with urothelial carcinomas of the upper urinary tract and urinary bladder [30]. Using immunohistochemistry, these authors showed that IGFBP5 overexpression was significantly associated with advanced tumor stage, and that it was an independent predictor of poor disease-specific survival and metastasis-free survival. We report here that this over-expression in MIBC is also a marker of cell non-sensitivity to IGF1R inhibitor. Our results should increase interest in IGFBP5. Indeed, as part of the current focus of pharmacological research on the INSR/IGF1R pathway in the treatment of cancer, more than 100 clinical trials have already investigated INSR/IGF1R inhibition. Several studies testing anti-IGF1R monoclonal antibodies reported low toxicity, with major clinical responses in some cases [31–33]. Nevertheless, the first phase III trial, which studied the addition of figitumumab to standard chemotherapy in the treatment of non-small-cell lung carcinoma was stopped after the inclusion of 682 patients because of toxicity, the signs of which included hyperglycemia in particular, together with a lack of antitumor efficacy [32]. The results of other phase III studies are expected soon, but schisms are already evident: some teams have abandoned the development of treatments targeting INSR/IGF1R, whereas others are continuing to study these receptors, whilst searching, in parallel, for biomarkers of the effectiveness of these therapies. Our results suggest that IGFBP5 expression could be used in this way in the bladder cancer setting.

Our study is the first on bladder cancer to use the public data from The Broad-Novartis Cancer Cell Line Encyclopedia (CCLE) collaborative project. This use of this database minimizes the methodological limitations relating to the reproducibility of proliferation inhibition assays. The results obtained are strengthened by the use of a validated registry for both gene expression and functional assays. On the down side, not all the data were obtained from cell lines of the same origin. Nevertheless, interlaboratory variability should not exceed intralaboratory variability (e.g. for high passage numbers) for specific cell lines [34].

## Conclusions

We found correlations between *IGFBP5* expression, the activation of the INSR/IGF1R receptors and the antiproliferative efficacy of an inhibitor of the IGF1R tyrosine kinase in bladder cancer derived cell lines suggesting that low expression of IGFBP5 could be used as a marker to predict anti-IGF1R or anti-IGF1R ligands therapies in bladder cancer. By contrast, the expression of IGF receptors and ligands was not correlated with these factors in bladder cancer samples and cell lines. Further investigations of the mechanisms by which IGFBPs interfere with bladder cancer oncogenesis are therefore required.

## Additional files

Additional file 1: Table S1. mRNA expression levels of the components of IGF pathway in the FBLAD-Exon set of 125 bladder tumors. Data were obtained from Human Genome Exon 1.0ST arrays. (XLS 27 kb)

Additional file 2: Figure S1. Levels of mRNA for IGF receptors, ligands, and binding proteins, by tumor stage, in the FLBAD-U95 dataset. *: *p* value = 0.01 to 0.05 **: *p* value = 0.001 to 0.01 ***: *p* value ≤0.001. (TIFF 24400 kb)

Additional file 3: Figure S2. IGF1R mRNA levels according to tumor stages in two independent publicly available data sets. (TIFF 7830 kb)

Additional file 4: Figure S3. IGF1R expression by epithelial cells in normal urothelium and bladder tumors. (a)Anti-IGF1R immunohistochemistry from human protein atlas project (http://www.proteinatlas.org/). 3 examples of representative staining in tumors are presented in the right panel, staining of the two normal samples are presented in the left panel. Scale bar represents 100 μm (b) Haematoxylin-eosin staining of our CIT-series of tumors. Examples of tumors with high and low IGF1R expression assessed by RPPA. (TIFF 7100 kb)

## Abbreviations

CCLE: Cancer Cell Line Encyclopedia
DNA: Deoxyribonucleic acid
IGF: Insulin growth factor
IGFBP: Insulin growth factor binding protein
IGFR: Insulin growth factor receptor
INS: Insulin
INSR: Insulin receptor
LIMMA: Linear Models for Microarray Data
MIBC: Muscle-invasive
mRNA: Messenger ribonucleic acid
NMIBC: Non muscle-invasive bladder cancer
RNA: Ribonucleic acid
RPPA: Reverse-phase protein array
