# Supplementary figures and images for: IGF1R activation and the in vitro antiproliferative efficacy of IGF1R inhibitor are inversely correlated with IGFBP5 expression in bladder cancer
Source: BMC Cancer. 2017 Sep 7;17:636. doi: 10.1186/s12885-017-3618-5 (PMC5588742; doi:10.1186/s12885-017-3618-5)

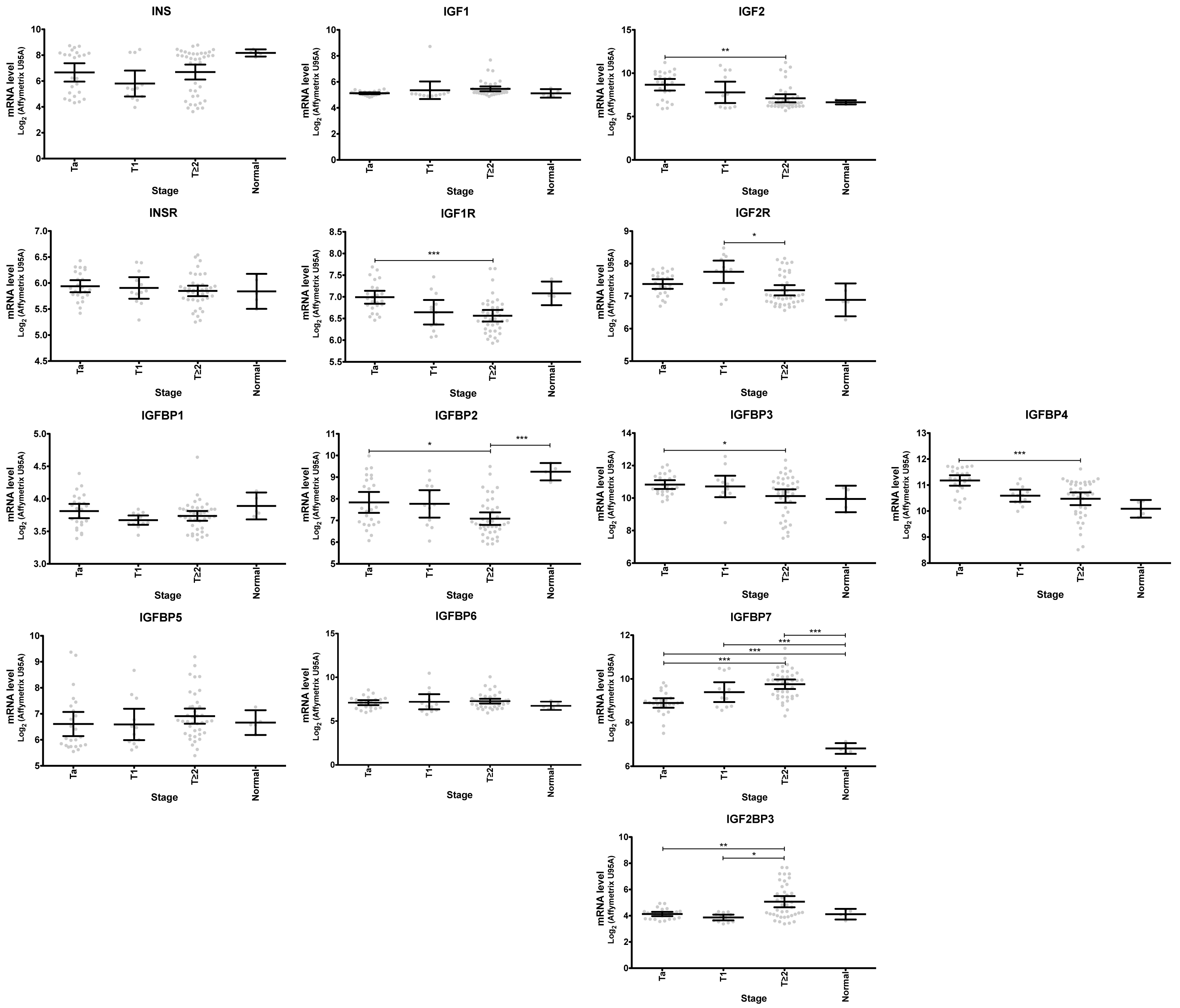

Supplement: Supplementary file 2 — Levels of mRNA for IGF receptors, ligands, and binding proteins, by tumor stage, in the FLBAD-U95 dataset. *: p value = 0.01 to 0.05 **: p value = 0.001 to 0.01 ***: p value ≤0.001. (TIFF 24400 kb) [file 12885_2017_3618_MOESM2_ESM.tif]

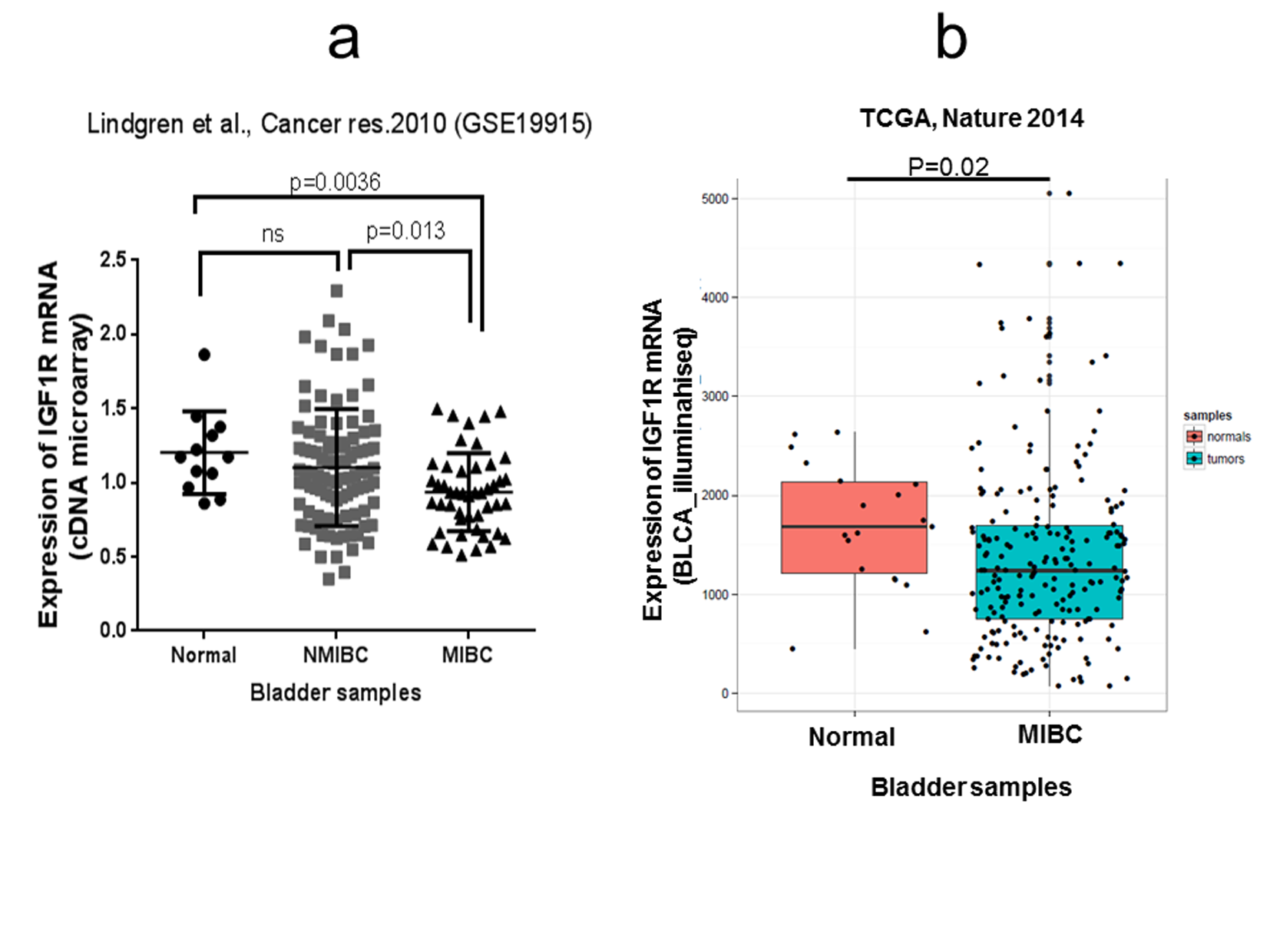

Supplement: Supplementary file 3 — IGF1R mRNA levels according to tumor stages in two independent publicly available data sets. (TIFF 7830 kb) [file 12885_2017_3618_MOESM3_ESM.tif]

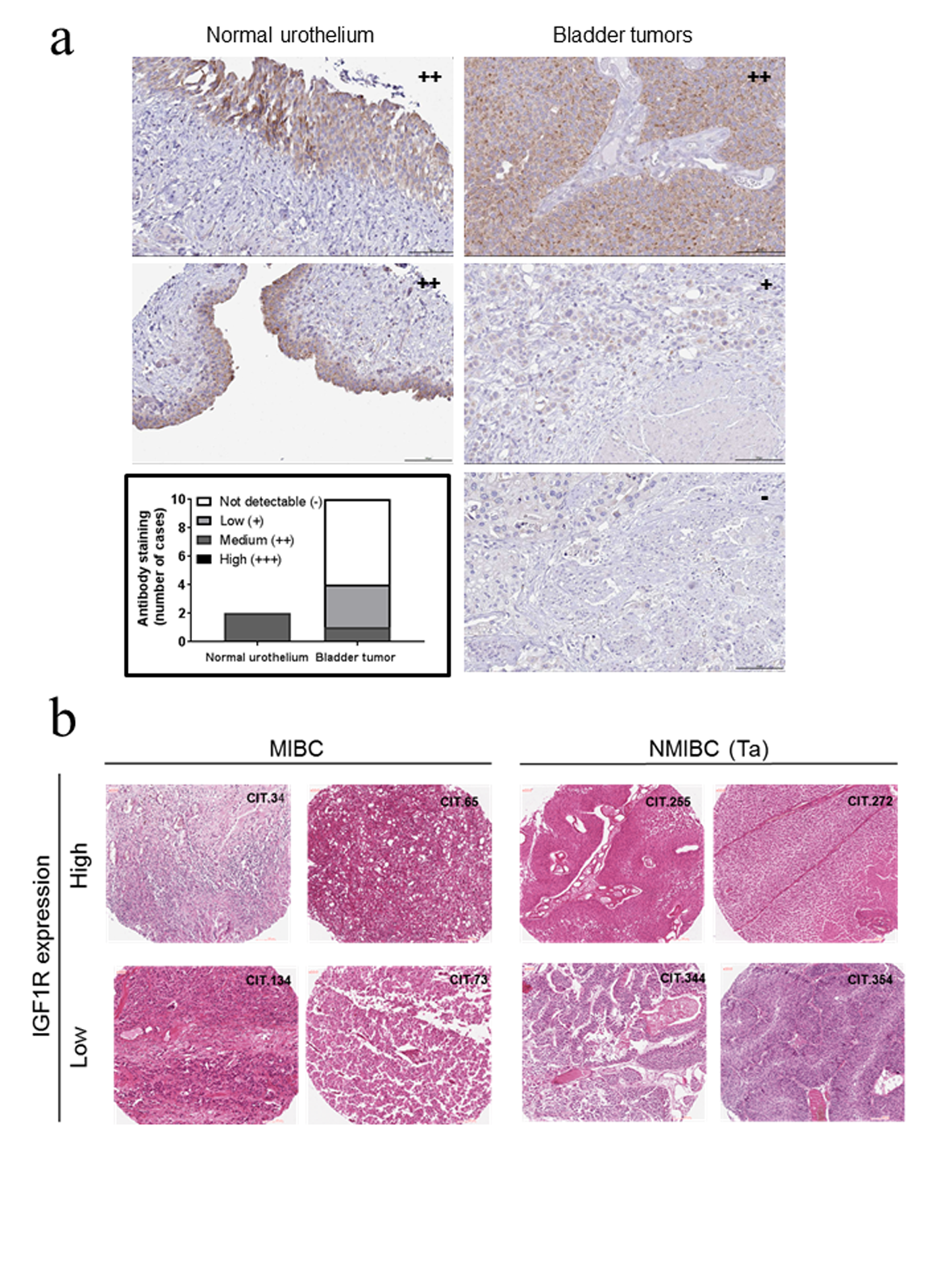

Supplement: Supplementary file 4 — IGF1R expression by epithelial cells in normal urothelium and bladder tumors. (a)Anti-IGF1R immunohistochemistry from human protein atlas project (http://www.proteinatlas.org/). 3 examples of representative staining in tumors are presented in the right panel, staining of the two normal samples are presented in the left panel. Scale bar represents 100 μm (b) Haematoxylin-eosin staining of our CIT-series of tumors. Examples of tumors with high and low IGF1R expression assessed by RPPA. (TIFF 7100 kb) [file 12885_2017_3618_MOESM4_ESM.tif]
